# Supplementary material for: Morphology Formation Pathways in Solution‐Processed Perovskite Thin Films
Source: Adv Sci (Weinh). 2025 Oct 21;13(5):e16701. doi: 10.1002/advs.202516701 (PMC12850291; doi:10.1002/advs.202516701)
Supplement: Supplementary file 1 — Supporting Information [file ADVS-13-e16701-s001.pdf]

Morphology Formation Pathways in Solution-Processed Perovskite Thin Films - Supporting Information

Martin Majewski,*[a] Olivier J.J. Ronsin,[b]‡ and Jens Harting[a,b]

[a] *Helmholtz Institute Erlangen-Nürnberg for Renewable Energy (HIERN), Forschungszentrum Jülich GmbH*
[b] *Department of Chemical and Biological Engineering and Department of Physics, Friedrich-Alexander-Universität Erlangen-Nürnberg, Fürther Straße 248, 90429 Nürnberg, Germany*

1 Introduction

In the following, an analytical model is created that can explain the different morphologies that are possible with a patterned substrate with two placed crystals and no further nucleation.
The model is based on the following assumptions:

- Direct crystallization is considered only, no SSP or intermediates are present in the system
- Only two dimensions are considered
- We start initially with two spherical crystals, equidistantly placed
- No further nucleation is considered
- The growth rate of the crystals (1D interfacial velocity) is constant and drops to zero when no solute in the liquid film is left or the liquid film is consumed completely.
- The crystals grow isotropically with a growth rate v_g unless there is no solute anymore in the liquid film, the crystal surface is in direct contact with the air or another crystal.
- The evaporation rate ($v_e = \frac{\text{d(height of the condensed film)}}{\text{dt}}$) is constant and drops to zero when there is no solvent left
- Solvent evaporates at the liquid-vapor interface, not at the solid-vapor interface.

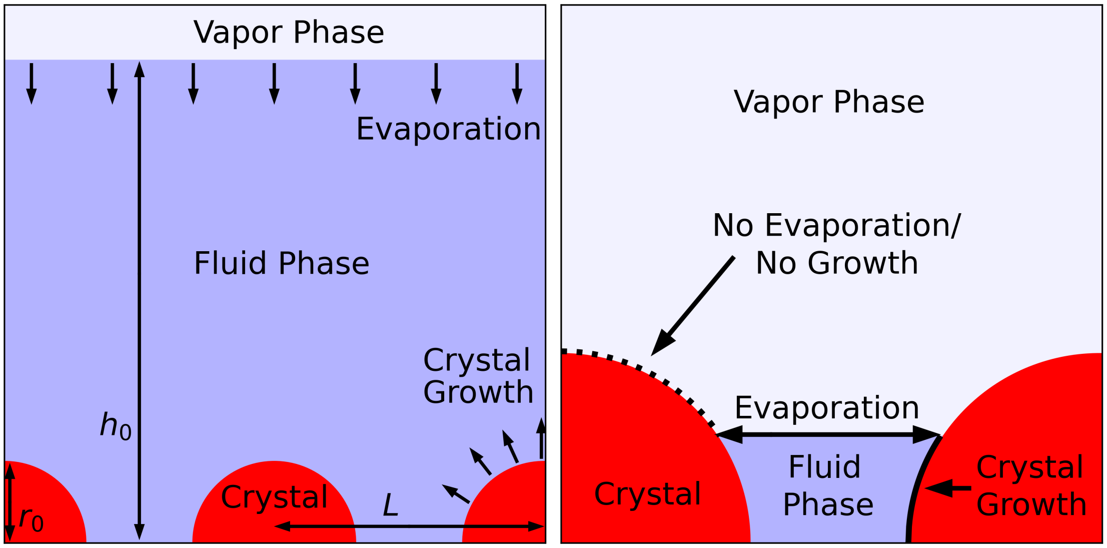

Fig. 1 Initial setup. The crystalline (red), fluid (dark blue) and vapor (light blue) phases are shown. L is the distance between the centers of the crystals, which is half of the width of the simulation box. The initial crystal radius r_{ini} and the initial wet film height h_{ini} are visualized.

1.1 Initial State

L is the distance between the crystal centers, hence the box width is $2L$. The initial film height is h_0, the radius of the two initially placed crystals is r_0, and the initial solute volume fraction is ϕ_0. The growth rate of the crystals is called v_g, and the evaporation rate of the solvent is called v_e.
The solute volume $V_{c,tot}$ is constant in time and can be calculated in the initial state via:

$$V_{c,tot} = 2L \cdot \phi_0 \cdot h_0 \tag{1}$$

The initial amount of solvent $V_{s,tot}$ is therefore

$$V_{s,tot} = 2L(1-\phi_0)h_0 \tag{2}$$

1.2 Dynamics

The average height h_f of the dry film is

$$h_f = V_{c,tot}/(2L) = h_0 \cdot \phi_0 \tag{3}$$

The time t_{max} it takes to evaporate the solvent completely is between maximally (if evaporation continues till the substrate)

$$t_{max} = h_0/v_e \tag{4}$$

for a setup with pinholes and t_{min} minimal

$$t_{min} = (h_0 - h_f)/v_e \tag{5}$$

for a final film that is completely flat.

2 Possible Final Film Structures

In the following, the classification described in sec 2 of the main text, is derived analytically. The considered configuration space is the ratio of growth to evaporation rate v_e/v_g and height of the initial wet film compared to the distance between the crystals L/h_{ini}.

2.1 Evaporation is finished when the crystal touches the surface (E$\rightarrow$ T, Sequence Nr. 1,2,3), purple

Evaporation is finished before the crystal reach the liquid film surface:

$$t_{min} < (h_f - r_0)/v_g \tag{6}$$

where r_0 is the initial crystal radius. Using Equation 5 this leads to

$$\frac{v_e}{v_g} > \frac{h_0 - h_f}{h_f - r_0} = \frac{h_0(1-\phi_0)}{h_0\phi_0 - r_0} \tag{7}$$

There we can distinguish three cases.
ETIG/Nr.1. The crystals grow up to the surface first and then impinge

$$h_f - r_0 < \frac{L}{2} - r_0 \tag{8}$$

and therefore (using Equation 3) this phase is restricted to

$$\frac{L}{h_0} > 2\phi_0 \tag{9}$$

IETG/Nr.2: The evaporation is finished earlier than the event of impingement:
Time for impingement:

$$t_i = \frac{L/2 - r_0}{v_g} \tag{10}$$

The time for evaporation is t_{min}. We solve for $t_{min} < t_i$:

$$\frac{v_e}{v_g} < \frac{h_0 - h_f}{L/2 - r_0} \tag{11}$$

EITG/Nr.3 is placed in-between these two domains.

2.2 Crystal growth terminates before the crystal touches the surface (G $\rightarrow$ T, Sequence Nr. 4,5), dark blue and dark yellow

There we can distinguish two cases depending on whether the crystals impinge in the final state (IGTE, Nr.5) or not (GTEI, Nr. 3).

These two phases are separated by:

$$L/2 = r_f \tag{12}$$

where r_f is the maximal extension of the crystal:

$$r_f = r_0 + v_g \cdot t_{growth} \tag{13}$$

with t_{growth} being the time for which all solute is consumed by growth. This time can be calculated from

$$V_c(t_{growth}) = V_{c,tot} \tag{14}$$

where $V_{c,tot}$ is the total amount of solute from Equation 1 and $V_c(t)$ is the time dependent volume of the two (half) crystals:

$$V_c(t) = r(t)^2 \cdot \pi = (r_0 + v_g t)^2 \cdot \pi \tag{15}$$

Inserting Equation 15 into Equation 14 and using Equation 1, leads to

$$t_{growth} = \frac{1}{v_g}\left(\sqrt{\frac{2h_0 L\phi_0}{\pi}} - r_0\right) \tag{16}$$

Inserting this into Equation 13 and Equation 12 (and neglecting the option of $L = 0$) results in:

$$\frac{L}{h_0} = \frac{8\phi_0}{\pi} \tag{17}$$

GTE, Nr.4: When the crystals do not impinge (dark blue), the condition for growth ending before the crystal touches the surface reads:

$$r(t_{growth}) < h(t_{grwoth}) \tag{18}$$

which can be rewritten as:

$$r_0 + v_g \cdot t_{growth} = h_0 - v_e \cdot t_{growth} \tag{19}$$

where t_{growth} is calculated by Equation 16. Inserting and rearranging results in

$$\frac{v_e}{v_g} < \frac{h_0 - \sqrt{\frac{h_0 2L\phi_0}{\pi}}}{\sqrt{\frac{h_0 2L\phi_0}{\pi}} - r_0} \tag{20}$$

IGTE, Nr. 5: In the situation where the crystal impinge, we have (dark yellow):

$$r(t_{growth}) \geq L/2 \tag{21}$$

To get an upper limit for v_e/v_g we need to calculate the Area of the grown crystals. The height of the grown crystals h_{max} is connected to the time of crystal growth t_{growth} by

$$h_{max} = r_0 + v_g \cdot t_{growth} \tag{22}$$

Let the height of the crystals at the grain boundaries be l_h. The area of one crystal consists of a circular segment A_2 and two triangles A_1 (see Figure 3 for definition of A_1 and A_2). l_h can be calculated in general as:

$$l_h = \sqrt{h_{max}^2 - (L/2)^2} \tag{23}$$

so that the size of the two triangles (together) is

$$A_1 = \frac{L}{2} \cdot \sqrt{h_{max}^2 - (L/2)^2} \tag{24}$$

The angle of the circular segment is

$$\alpha = 2\arcsin\left(\frac{L}{2h_{max}}\right) \tag{25}$$

so that the size of the area is then

$$A_2 = h_{max}^2 \arcsin\left(\frac{L}{2h_{max}}\right) \tag{26}$$

Gathering Equation 24 and Equation 26 the crystalline volume in the final state V_{cryst} is

$$V_{cryst} = 2(A_1 + A_2) = \frac{L}{2} \cdot \sqrt{h_{max}^2 - (L/2)^2} + h_{max}^2 \arcsin\left(\frac{L}{2h_{max}}\right) \tag{27}$$

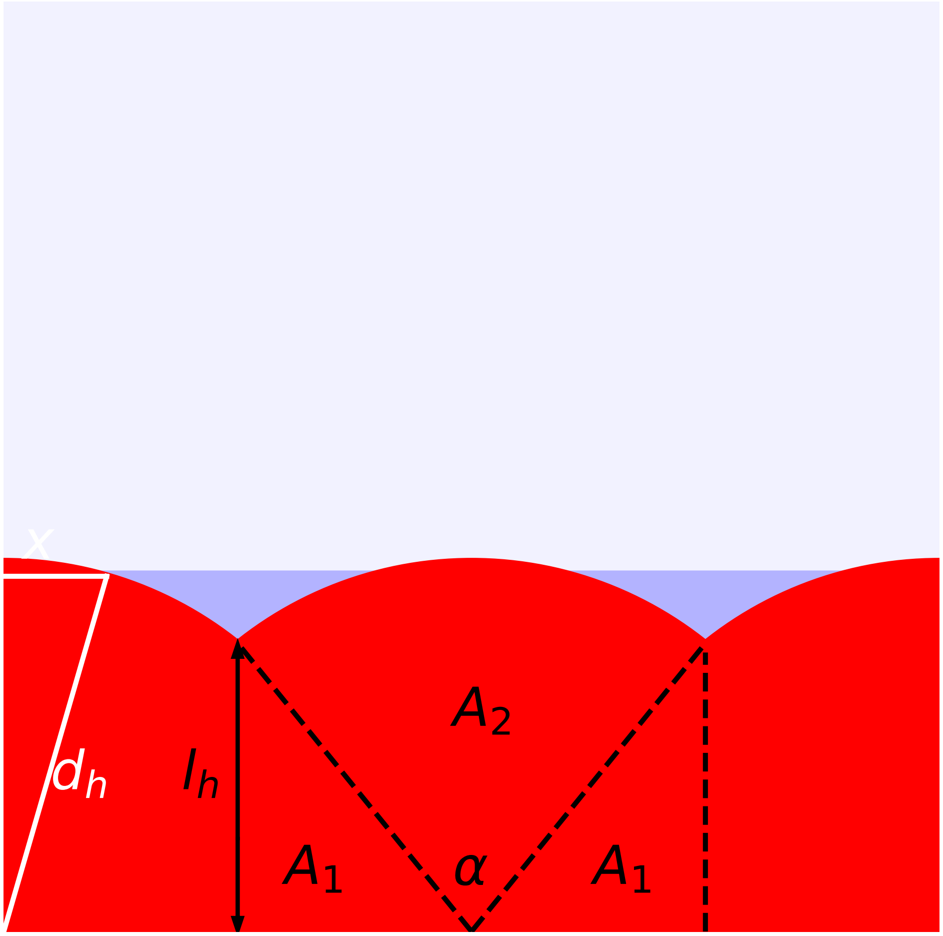

Fig. 2 Intermediate stage. The crystalline (red), fluid (light blue) and vapor (dark blue) phases are shown. Shown are the areas A_1 and A_2, the lengths l_h, h_{max} and x, and the angle α

At the end of the crsytallization process, we have $V_{cryst} = V_{c,tot}$. Using Equation 1, h_{max} can be found by solving numerically

$$2(A_1 + A_2) = \frac{L}{2} \cdot \sqrt{h_{max}^2 - (L/2)^2} + h_{max}^2 \arcsin\left(\frac{L}{2h_{max}}\right) = 2L \cdot \phi_0 \cdot h_0 \tag{28}$$

The condition for growth finishing before the crystal touches the surface corresponds to the top of the crystal reaching the position h_{max} before the film surface:

$$\frac{h_{max} - r_0}{v_g} = \frac{h_0 - h_{max}}{v_e} \tag{29}$$

which we rewrite as:

$$\frac{v_e}{v_g} < \frac{h_0 - h_{max}}{h_{max} - r_0} \tag{30}$$

2.3 The crystals touch the surface.

For all the other cases neither crystal growth nor evaporation is finished when the film surface touches the crystals. The time t_T of for the first contact between crystal and film surface is defined by calculating the crystal radius r_T at t_T via two different ways :

$$r_T = r_0 + v_g t_T = h_0 - v_e t_T \tag{31}$$

and therefore

$$t_T = (h_0 - r_0)/(v_g + v_e) \tag{32}$$

The volume of the crystals at the time when they com in contact with the film surface is:

$$V_T = \pi(r_0 + t_T \cdot v_g)^2 \tag{33}$$

Once the crystal and film surfaces have touched each other, growth can terminate either before or after evaporation. To investigate this transition, we can have a look at the solvent in the system:

The amount of solvent left at t_T is

$$V_{s,T} = V_{s,tot} - v_e \cdot t_T \cdot 2L \tag{34}$$

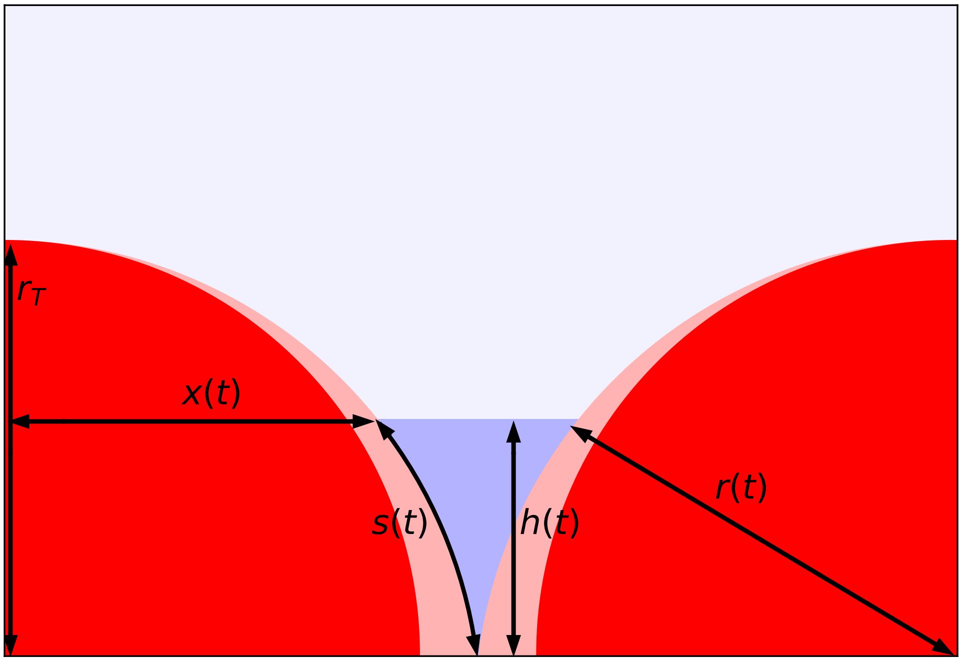

Fig. 3 Sketch of an intermediate stage. The crystalline (light red), fluid (light blue) and vapor (dark blue) phases are shown. A circle is displayed in dark red inside the crystal for comparison. Shown are $x(t)$, r_T, $r(t)$, $h(t)$ and $s(t)$.

The amount of solvent reduces after t_T according to

$$V_s(t) = V_{s,T} - \int_{t_T}^{t} dt\,(v_e \cdot (2L - 4x(t)) = 2L(1-\phi_0)h_0 - v_e \cdot t_T \cdot 2L - \int_{t_T}^{t} dt\,(v_e \cdot (2L - 4x(t))) \tag{35}$$

where $x(t)$ is half of the width of a crystal at the height of the liquid-vapor interface (compare Figure 3)

$$x = \sqrt{r^2(t) - h^2(t)} = \sqrt{(r_0 + v_g t)^2 - (h_0 - v_e \cdot t)^2} \tag{36}$$

Note that the calculation of V_s bases on the size of the liquid-vapor interface only, Equation 35 holds, no matter what happens regarding impingement. By definition we have

$$V_s(t_s) = 0 \tag{37}$$

In addition, this corresponds to the situation where evaporation stops exactly when the size of the liquid-vapor interface drops to zero $(2L - 4x(t_s) = 0)$. Since:

$$\frac{d}{dt}V_s(t)\bigg|_{t_s} = v_e(2L - 4x(t_s)) \tag{38}$$

we have:

$$\frac{d}{dt}V_s(t)\bigg|_{t_s} = 0 \tag{39}$$

Therefore, searching for the boundary between regions where growth ends before evaporation and the vice versa, means searching for Equation 35 having its minimal value equal to zero. We solve this for v_e/v_g numerically for each L/h_0 value.

2.4 'Intermediate fast' evaporation: evaporation terminates before crystal growth (T $\rightarrow$ E $\rightarrow$ G, formation pathways Nr. 6, 7, 8, red

ITEG, Nr. 7 and TIEG, Nr. 8: Impingement can take place before or after the crystals touch the film surface. The time for impingement is given by Equation 10 and the time for crystal surface contact by Equation 32, so that the interface is exactly when:

$$\frac{L/2 - r_0}{v_g} = \frac{h_0 - r_0}{v_e + v_g} \tag{40}$$

Rearranging:

$$\frac{v_e}{v_g} = \frac{2h_0 - L}{L - 2r_0} \tag{41}$$

TEIG, Nr. 6 and TIEG, Nr. 8: Impingement may occur before or after evaporation terminates. The condition for the interface in between is $t_{evap} = t_i$ with t_i given by Equation 10, but this time $t_{evap} = t_s$ such that $V_s(t_s) = 0$ with, V_s defined by Equation 35. We numerically solve for v_e/v_g to find $t_s = t_i$ in order to find the boundary.

2.5 'Intermediate slow' evaporation: crystal growth terminates before evaporation (T $\rightarrow$ G $\rightarrow$ E, formation pathways Nr. 9, 10, 11), light blue, light and medium yellow

ITGE, Nr 10 and TIGE, Nr. 11: The boundary is equal to Equation 41.

TGE, Nr. 9: The crystals do not impinge each other, but the crystals still grow when the crystals touch the vapor (light blue).

After t_T the surface of the crystals that is still surrounded by liquid and where growth further proceed is reduced to $4s(t)$:

$$s(t) = r(t)\arcsin\left(\frac{h(t)}{r(t)}\right) = (r_0 + v_g t)\arcsin\left(\frac{h_0 - v_e \cdot t}{r(t)}\right) \tag{42}$$

Considering the 4 growing regions, the total crystalline volume $V_{crystal}(t)$ after t_T reads (as long neither growth nor evaporation terminate):

$$V_{crystal}(t) = V_T + \int_{t_T}^{t} dt\,(4 \cdot s(t) \cdot v_g) \tag{43}$$

At the end of crystal growth we have:

$$V_{crystal}(t_{max}) = V_{c,tot} \tag{44}$$

Now, growth terminates before the crystals can impinge if:

$$t_{maxgrowth} < \frac{1}{v_g}\left(\frac{L}{2} - r_0\right) \tag{45}$$

This means that the limiting condition for end of growth without impingement (film with pinholes) reads:

$$V_{crystal}\left(t = \frac{1}{v_g}\left(\frac{L}{2} - r_0\right)\right) = V_{sol,tot} \tag{46}$$

Using Equation 1 , Equation 32, Equation 33, Equation 42, Equation 43, Equation 46 this leads to:

$$\pi(r_0 + (h_0 - r_0)/(v_g + v_e) \cdot v_g)^2 + \int_{(h_0 - r_0)/(v_g + v_e)}^{t} dt\,(4 \cdot r(t)\arcsin\left(\frac{h_0 - v_e \cdot t}{r(t)}\right) \cdot v_g) = 2L(1 - \phi_0)h_0 \tag{47}$$

For all other parameters fixed, we numerically solve the equation above for v_e/v_g. This defines, for each L/h_0 value the upper limit on v_e/v_g for a film with pinholes.

2.6 Roughness

To calculate the roughness for the complete surface is too complicated. To get an approximation for it we evaluate the highest point of the dry film and compare it to the average film thickness.

$$R/h_0 = \frac{h_{max} - h_f}{h_0} \tag{48}$$

For the formation pathways 1-3 the roughness is zero. For the formation pathways 4 and 5 we take the calculated value of h_{max} (compare subsection 2.2) for the equation above. For the remaining pathways the maximal height of the film is defined by the time the crystals get in contact to the vapor phase. In these cases $h_{max} = r_T$ (comapre subsection 2.3).

2.7 Uncovered substrate

There can only be uncovered substrate if no impingement occurs. That leaves formation pathway 4 and 9. For the formation pathway 4 the amount of uncovered substrate can be calculated by

$$S/L = \frac{L - 2 \cdot r(t_{growth})}{L} \tag{49}$$

t_{growth} being the crystal radius when the crystal growth terminates, defined in subsection 2.2.

For formation pathway 9 we can calculate $r(t_{max})$ and the amount of uncovered substrate as above (compare subsection 2.5).

3 Boundary representations with roughness and the amount of uncovered substrate.

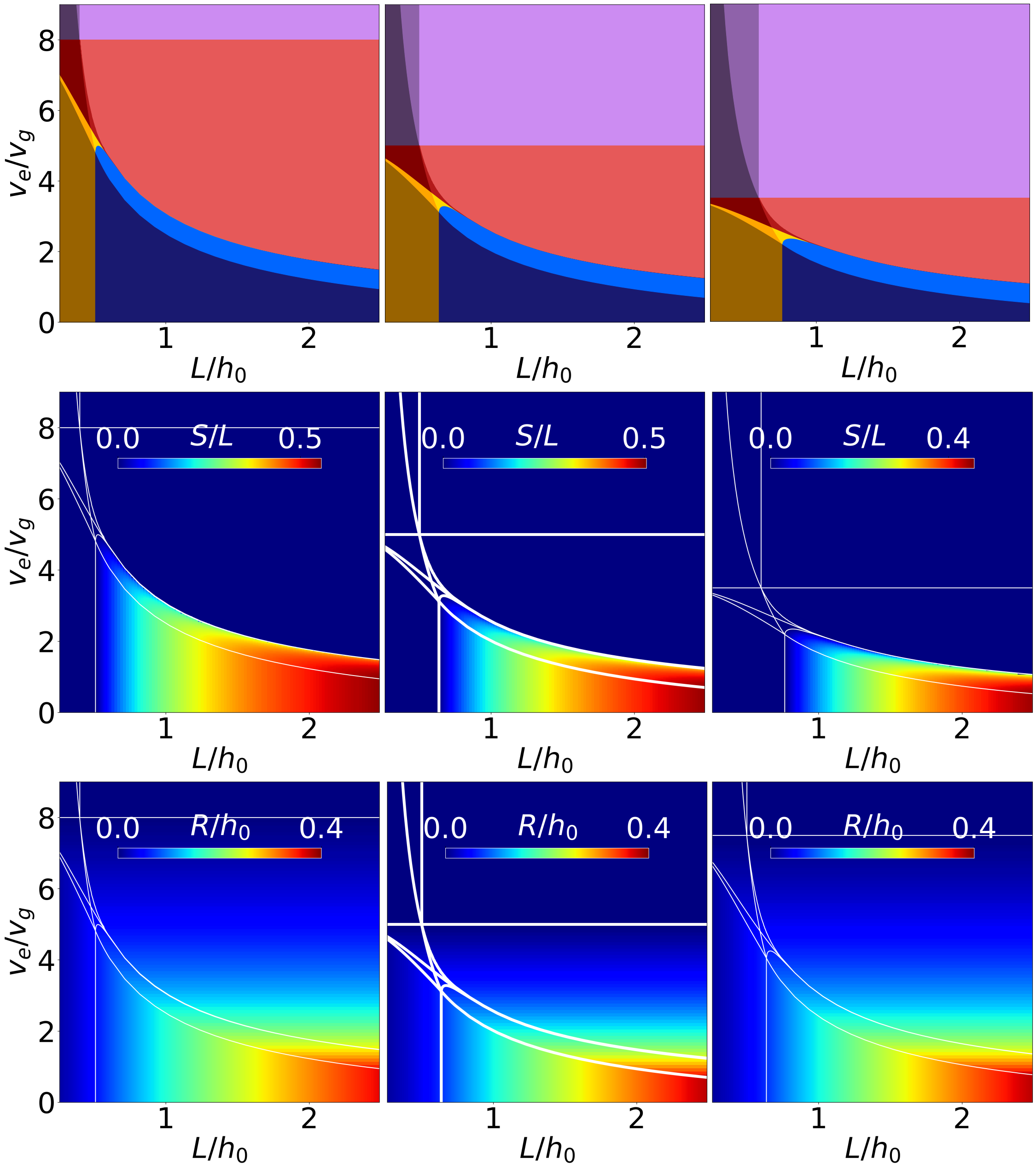

Fig. 4 Initial volume fraction (ϕ_0) variation. ϕ_0 increases from left to right (ϕ_0 = 0.2, 0.25, 0.3). The initial ratio of crystal size to film height is $r_0/h_0 = 0.1$. The first row are the boundary representations from the main text. The second and third rows are the corresponding amount of uncovered substrate and roughness plots.

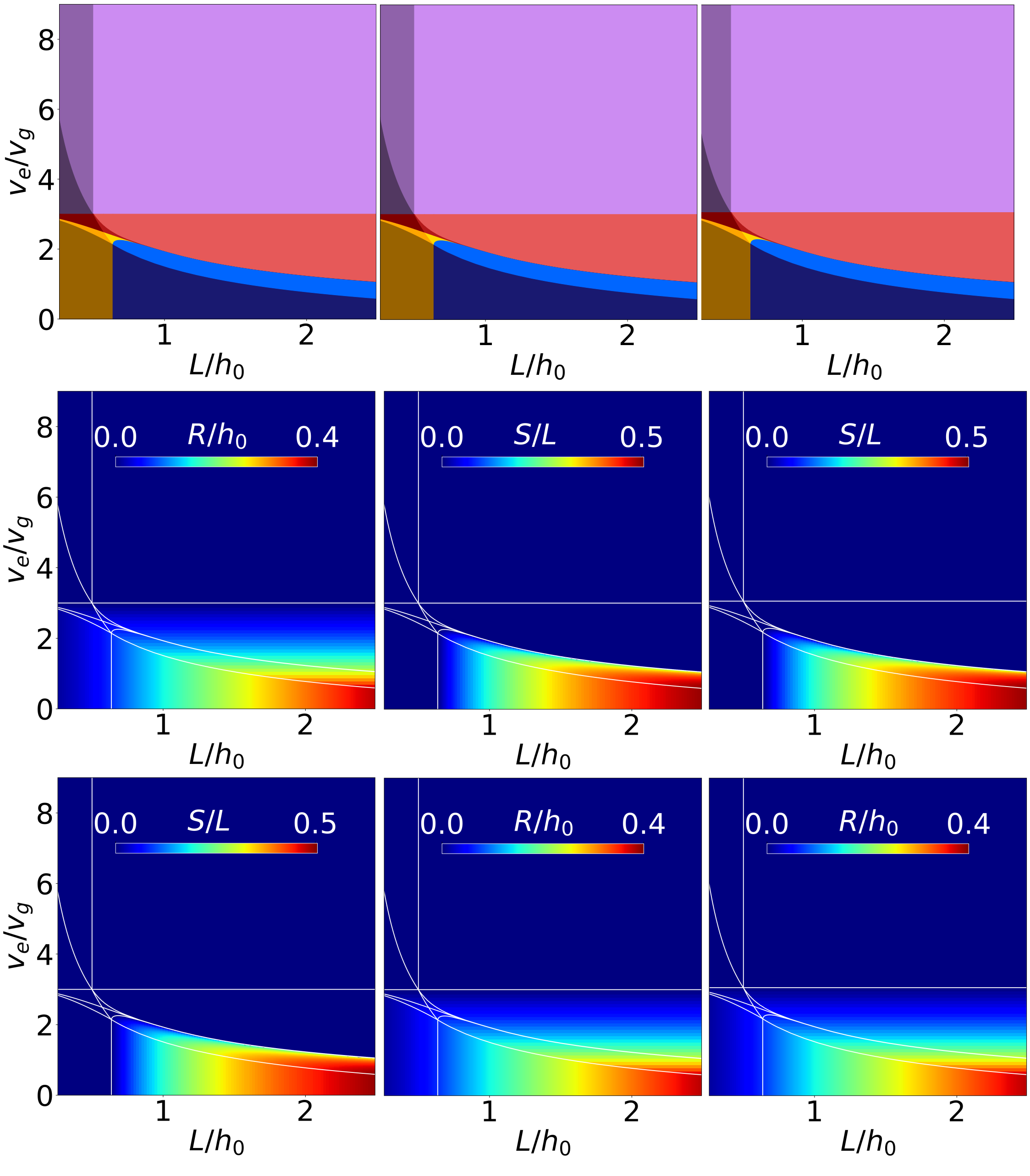

Fig. 5 Initial crystal size variation. The ratio r_0/h_0 increases from left to right ($r_0/h_0 = 5 \cdot 10^{-5}$, $5 \cdot 10^{-4}$, $5 \cdot 10^{-3}$). The initial volume fraction is $\phi_0 = 0.25$. The first row is the boundary representations from the first row of figure 5 in the main text. The second and third row are the corresponding uncovered substrate and roughness plots.

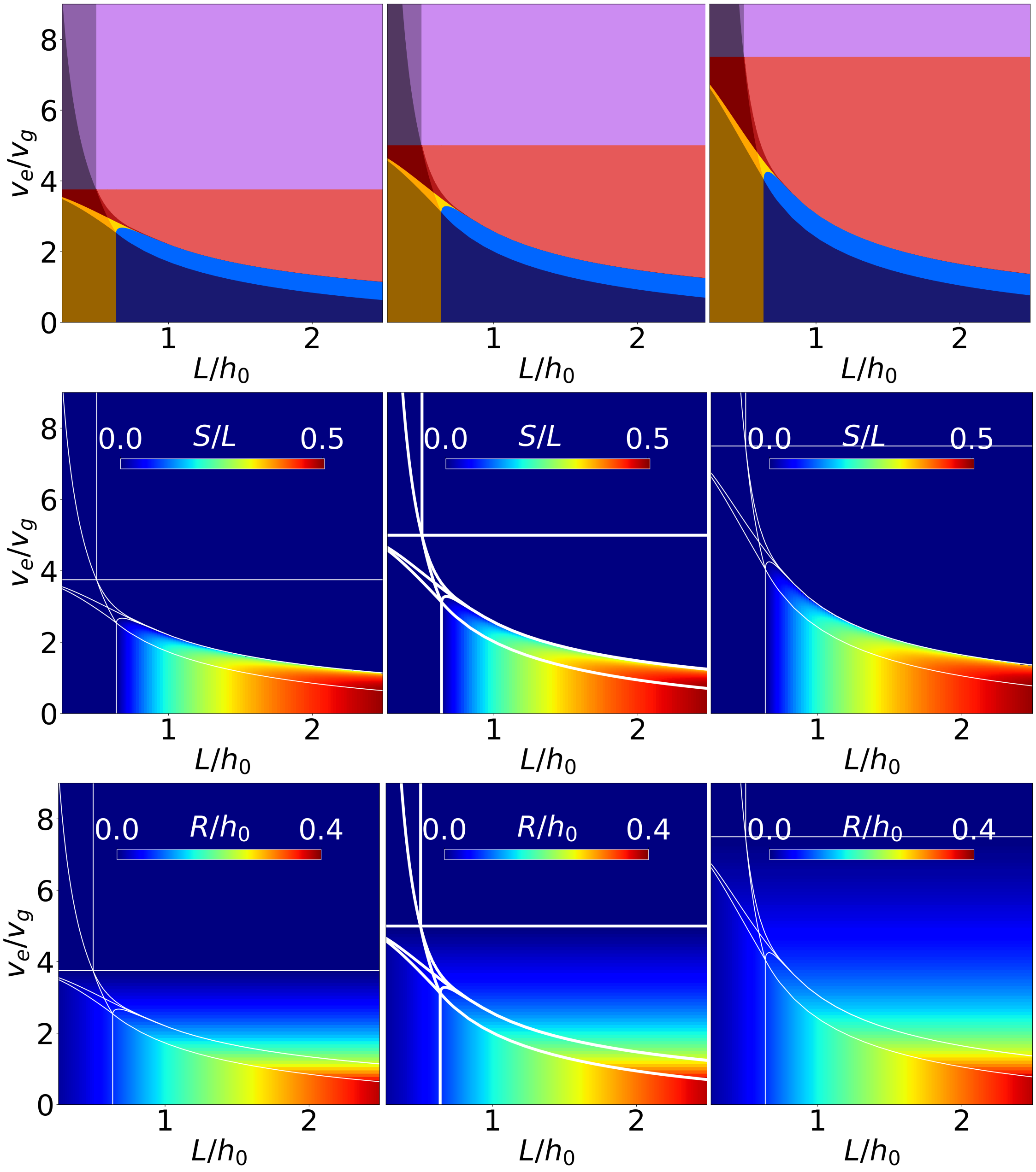

Fig. 6 Initial crystal size variation. The ratio r_0/h_0 increases from left to right (r_0/h_0 = 0.05, 0.1, 0.15). The initial volume fraction is $\phi_0 = 0.25$. The first row is the boundary representations from the second row of figure 5 in the main text. The second and third row are the corresponding uncovered substrate and roughness plots.

4 Changing the initial film height

In this section, we discuss the change of the initial film height. In the model, the initial film height is used as a scaling parameter for the x-axis. Additionally, the ratio of initial crystal size to initial film height defines the shape of the boundary representation (compare section 5, main text). For very small ratios of r_0/h_0, the effect of changing the ratio is barely visible (compare Figure 5). Hence, changing only the initial film height will lead to a horizontal displacement in the boundary representation.

For larger values of r_0/h_0, the picture is more complicated. For example: Increasing h_0 by a factor of two is equivalent to dividing the initial value of L/h_0 by two and could be equivalent to changing the central boundary representation in Figure 6 to the left one. So increasing the film height may (depending on the starting point) move the formation pathway from a film with pinholes to one without (by the horizontal displacement) or it may turn a rough film to a fully smooth one (by the vertical displacement of the interfaces due to the changed boundary representation).

From a practical perspective, a higher initial film height leads to a higher final film height[1], which may increase light absorption and therefore enhance efficiency[2]. The film height can easily be changed for seeded growth as well as heterogeneous nucleation on the

substrate. Surprisingly, the necessary evaporation rate to obtain a smooth film (purple region) decreases for an increasing film thickness.

5 Simulation model

In this section the equations describing the evolution in the simulation are described.

5.1 Gibbs Free Energy

The simulation setup is a simplified version of[3]. The system is modeled with three components: the crystallizing material (the solute, φ_1), the evaporating material (solvent, φ_2), and the buffer material (air, φ_3) replacing the solvent. Additionally, the system contains two order parameters to define, whether it is in a fluid state (where both order parameters are zero), in a crystalline state (where the crystalline order parameter $\phi_c = 1$), or in a vapor state (where the vapor order parameter $\phi_{air} = 1$). Finally, there is a labeling field θ to handle the polycristallinity of the film.

The energy of the system is integrated over the whole system V and is given by the Gibbs free energy G

$$G = \int_V \left(\Delta G^{local} + \Delta G^{interf} \right) dV \tag{50}$$

that can be split into a local contribution ΔG^{local} and a part, that accounts for the surface tensions arising from the interfaces in the system ΔG^{interf}.

The local contribution can be split into

$$\Delta G^{local} = \left(1 - \phi_{air}^2(3 - 2\phi_{air})\right) \Delta G^{condensed} + \Delta G^{FH} + \phi_{air}^2(3 - 2\phi_{air})\Delta G^{air} + \Delta G^{numerical} \tag{51}$$

where the condensed part $\Delta G^{condensed}$ accounts for the energy difference between the liquid and the solid state, ΔG^{FH} accounts for the entropic and intramolecular interactions, modeled by the Florry Huggins theory. ΔG^{air} models the vapor part of the system and $\Delta G^{numerical}$ adds some contributions to enhance numerical stability. $\Delta G^{condensed}$ can be written as:

$$\Delta G^{condensed} = \rho \varphi_1^2 \left(\phi_c^2 (1 - \phi_c)^2 W + \phi_c^2 (3 - 2\phi_c) \Delta G^{cryst} \right) \tag{52}$$

where ρ is the density of the materials, W defines the energy barrier between fluid and solid state, and $\Delta G^{cryst} = L(T/T_m - 1)$, with L being the enthalpy of fusion, T the temperature and T_m the melting temperature of the perovskite.

The intramolecular interactions between the species ΔG^{FH} can be written as

$$\Delta G^{FH} = \frac{RT}{v_0} \left(\sum_{i=1}^{3} \varphi_i ln(\varphi_i) + \sum_{i=1}^{3} \sum_{j>i}^{3} \varphi_i \varphi_j \chi_{ij,LL} + \sum_{j=2}^{3} \phi_c^2 \varphi_1 \varphi_j \chi_{1j,SL} \right) \tag{53}$$

where R is the gas constant, v_0 is the volume of the smallest lattice site, as defined in the Florry Huggins theory, $\chi_{ij,LL}$ accounts for the interactions between material φ_i and material φ_j and $\chi_{1j,SL}$ correspondingly between the solid part of material φ_1 and material φ_j.

The energy of the air is modeled as an ideal gas:

$$\Delta G^{air} = \frac{RT}{v_0} \sum_{i=1}^{3} \varphi_i ln \left(\frac{\varphi_i}{\varphi_{sat,i}} \right) \tag{54}$$

where $\varphi_{sat,i} = P_{sat,i}/P_0$ with $P_{sat,i}$ being the vapor pressure of material φ_i and P_0 a reference pressure. The numerical contribution can be written as:

$$\Delta G^{numerical} = E_0 \frac{d_{sv}}{f(\varphi_i \phi_c, d_{sv}, c_{sv}, w_{sv})} + \sum_{i=1}^{3} \frac{\beta}{\varphi_i} \tag{55}$$

where the first summand prevents the crystalline and the vapor phase from penetrating each other, with E_0 defining the strength of the penalty and f being an interpolating function and d_{sv}, c_{sv}, w_{sv} being its amplitude, center, and width as defined below. The second summand helps to increase the possible step size of the simulation by preventing the volume fractions from becoming too small, where β defines the strength of this penalty and it is chosen small enough to have negligible impact on the physical properties. The interpolating function is defined as

$$\log f(x, d, c, w) = \frac{1}{2} \log(d)(1 + \tanh(w(x - c))) \tag{56}$$

The surface tension arises from the interfacial contribution

$$\Delta G^{interf} = \sum_{i=1}^{3} \frac{\kappa_i}{2} (\nabla \varphi_i)^2 + \frac{\varepsilon_{air}^2}{2} (\nabla \phi_{air})^2 + \frac{\varepsilon_c^2}{2} (\nabla \phi_c)^2 + p(\phi_c) \frac{\pi \varepsilon_g}{2} \delta (\nabla \theta) \tag{57}$$

where κ_i defines the surface tension arising from volume fraction variations of material φ_i, ε_{air} defining the contribution of the interface between the vapor and non-vapor phase, ε_c defining the contribution arising from the crystalline - noncrystalline interface, and ε_g

accounting for the energy contribution of crystal boundaries.

5.2 Allen Cahn and Cahn Hilliard Equation

The evolution of the volume fractions is modeled by the Cahn-Hilliard equation

$$\frac{\partial \varphi_i}{\partial t} = \frac{v_0}{RT} \nabla \left[\sum_{j=1}^{2} \Lambda_{ij} \nabla(\mu_j - \mu_3) \right] \tag{58}$$

where Λ_{ij} are the onsager mobilities and $\nabla(\mu_j - \mu_3)$ is the difference in chemical potential. The evolution of the order parameters is given by the Allen Cahn equation

$$\frac{\partial \phi_i}{\partial t} = -\frac{v_0}{RT} M_i \frac{\delta \Delta G}{\delta \phi_i} \tag{59}$$

where i stands for *air* or c for the vapor or crystalline phase respectively, and M_i is the respective Allen Cahn mobility. The evaporation of the solvent is modeled as an outflux j at the top of the simulation box $z = z_{max}$

$$j^{z=z_{max}} = \alpha \sqrt{\frac{v_0}{2\pi RT\rho}} P_0(\varphi_2^{vap} - \varphi^{\infty}) \tag{60}$$

where α is the evaporation condensation coefficient and φ_2^{vap} is the vapor pressure of the solvent and φ^{∞} the vapor pressure of the environment. It was ensured that this outflux reproduces the evaporation rates measured in the experiment[4].

6 Parameters (Simulation)

Table 1 Parameters of the simulations

Parameters	Full Name	Value	Unit
α	Evaporation-condensation-coefficient	$2.3 \cdot 10^{-5}$	-
dx, dy	Grid Spacing	1, 1	nm
$\phi_{1,ini}$	Initial precursor concentration	0.20, 0.25 or 0.3	-
nx	Grid Size in horizontal direction	160, 192, 224, 256, 288, 320, 352, 384, 416	-
ny	Grid Size in vertical direction	256	-
T	Temperature	300	K
ρ	Density	1000	kg/m^3
m	Molar Mass	0.1, 0.1, 0.03	kg/mol
v_0	Molar Volume of the Florry Huggins Lattice Site	$3 \cdot 10^{-5}$	m^3/mol
$\chi_{12,LL}, \chi_{13,LL}, \chi_{23,LL}$	Liquid - liquid interaction parameter	0.57, 1, 0	-
$\chi_{12,SL}, \chi_{13,SL}$	Liquid - solid interaction parameter	0.15, 0.5	-
T_m	Melting Temperature	600	K
L_{fus}	Heat of Fusion	75789	J/kg
W	Energy barrier upon crystallization	142105	J/kg
P_0	Reference Pressure	10^5	Pa
$P_{sat,1}, P_{sat,2}, P_{sat,3}$	Vapor Pressure	10^2, $1.5 \cdot 10^4$, 10^8	Pa
P_i^∞	Partial Vapor Pressure in the Environment	0	Pa
E_0	Solid-Vapor interaction energy	$5 \cdot 10^9$	J/M^3
β	Numerical Free Energy Coefficient	10^{-5}	J/M^3
κ	Surface Tension Parameters for Volume Fraction Gradients	$6 \cdot 10^{-9}$ (all)	J/m
$\varepsilon_c, \varepsilon_{vap}$	Surface Tension Parameters for Order Parameter Gradients	$3 \cdot 10^{-5}$, 10^{-4}	$(J/m)^{0.5}$
$D_{s,i}^{\Phi_j \to 1}$	Self-Diffusion Coefficients in pure materials	10^{-9} (all)	m^2/s
M_c	Allen Cahn mobility coefficient for the crystalline phase	0.5, 0.75, 1, 1.5, 2, 2.5, 3	s^{-1}
M_v	Allen Cahn mobility coefficient for the vapor phase	10^6	s^{-1}
$D_1^{vap}, D_2^{vap}, D_3^{vap}$	Diffusion Coefficients in the Vapor Phase	10^{-18}, 10^{-10}, 10^{-10}	m^2/s
d_{sl}, c_{sl}, w_{sl}	Amplitude, center and with of the penalty function for the diffusion coefficients upon liquid solid transition	10^{-9}, 0.7, 10	-
d_{sv}, c_{sv}, w_{sv}	Amplitude, center and with of the penalty function for the Allen Cahn mobility and the solid- vapor interaction energy	10^{-9}, 0.3, 15	-
r_{ini}	Radius of the initially placed crystals	10, 20, 30	nm

7 Crystal Growth Rate in the Simulation

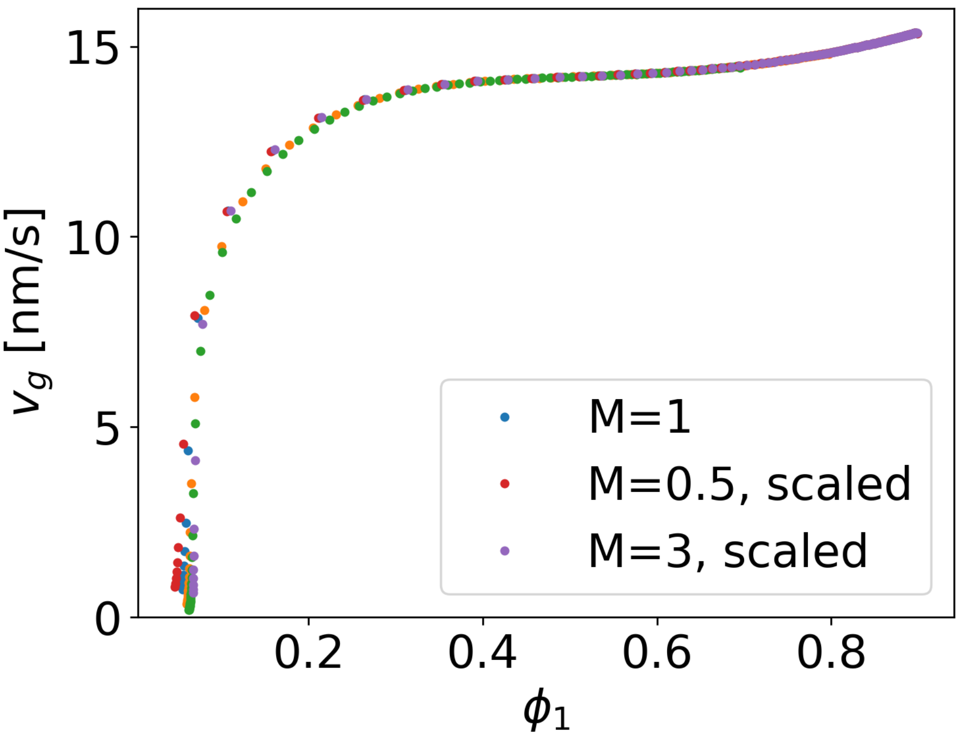

Fig. 7 Grow Rates depending on the solute volume fraction Φ_1 evaluated from a one-dimensional simulation with a singular crystal placed. Three different Allen-Cahn mobilities were tested to ensure consistency. The curves for M = 0.5 and M = 3 are scaled with 2 and 1/3.

8 Verification of the theoretical model against PF Simulations

The validity of the model is cross checked against a PF simulation[3]. The simulation takes into account the three components of solute, solvent and vapor, and captures evaporation, crystal growth and diffusion. The initial arrangement is the same as that used in the theoretical model. The main differences between the two approaches are: (1) the interfaces between the phases are sharp in the theoretical model but diffuse in the PF approach; (2) surface tensions play a role in the PF simulation; and (3) in the PF simulation the crystal growth rate and the solvent evaporation rate weakly depend on the surrounding volume fraction. Additionally, the crystalline phase consists a residue amount of solvent, and crystal growth stops at a volume fraction of a few percent solute (at the thermodynamic equilibrium).

The visual representation is shown in Figure 8. Between simulation runs/minimal model evaluations, we vary the ratio of evaporation to growth rate v_e/v_g and the distance between the crystals L. We scale these distance with the initial film height h_{ini} to obtain a dimensionless quantity. In Figure 8, simulations are represented by circles, and the background color indicates the morphology classification scheme. First, all the formation pathways can be recovered in the simulation. Additionally, there is a good agreement between theory and simulation, although slight deviations are observed. The reasons for these deviations are:

First, in the simulations, the growth rate is not constant for all volume fractions (see section 7). To partially compensate for this, the growth rate of the crystals is averaged from the initial to the dry state. This is achieved by tracking the composition in the wet film and averaging the interface velocities obtained from the 1D crystal growth simulations in section 7. Although this simple correction improves the agreement, it is not sufficient to fully compensate for all the differences with the minimal model. This leads to a slight inaccuracy in the value of v_e/v_g.

Second, in the Phase Field simulation, a surface tension between the different materials and phases must be included. This leads to a minimal possible gap distance larger than zero between the crystals. Consequently, the boundary for low v_e/v_g is shifted to larger L/h_0 values compared to the theoretical model. Additionally, the Phase Field simulation contains diffuse interfaces, also preventing very small gaps between the crystals (region of light blue).

Further effects that contribute to small deviation to the theory include: the volume fraction of every material at any point in the simulation cannot be exactly zero, and the evaporation rate is not perfectly constant in the simulation[4].

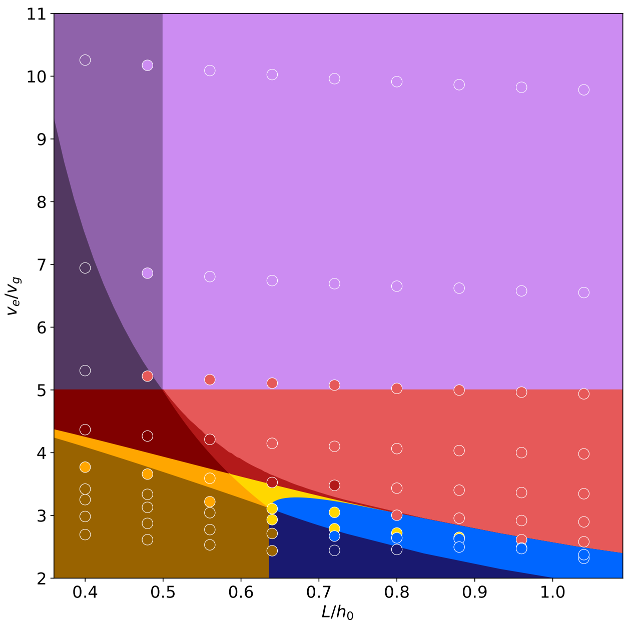

Fig. 8 Comparison between the model and the results of the Phase Field simulations. The initial volume fraction of crystalline material is $\phi_0 = 0.25$, and the initial ratio of crystal size to film height is $r_0/h_0 = 0.1$ (same as in Figure 2/3 in the main text). The simulations are represented as dots. There is an overall good agreement.

Notes and references

1 F. Gumpert, A. Janßen, C. J. Brabec, H.-J. Egelhaaf, J. Lohbreier and A. Distler, *Engineering Applications of Computational Fluid Mechanics*, 2023, **17**, 2242455.

2 T. Ouslimane, L. Et-taya, L. Elmaimouni and A. Benami, *Heliyon*, 2021, **7**, e06379.

3 O. J. J. Ronsin and J. Harting, *Advanced Theory and Simulations*, 2022, 2200286.

4 O. J. J. Ronsin, D. Jang, H.-J. Egelhaaf, C. J. Brabec and J. Harting, *ACS Applied Materials & Interfaces*, 2021, **13**, 55988–56003.
